# The Chromatin Remodeler SPLAYED Regulates Specific Stress Signaling Pathways

**DOI:** 10.1371/journal.ppat.1000237

**Published:** 2008-12-12

**Authors:** Justin W. Walley, Heather C. Rowe, Yanmei Xiao, E. Wassim Chehab, Daniel J. Kliebenstein, Doris Wagner, Katayoon Dehesh

**Affiliations:** 1 Department of Plant Biology, University of California, Davis, California, United States of America; 2 Department of Plant Sciences, University of California, Davis, United States of America; 3 Department of Biology, University of Pennsylvania, Philadelphia, Pennsylvania, United States of America; The University of North Carolina at Chapel Hill, United States of America

## Abstract

Organisms are continuously exposed to a myriad of environmental stresses. Central to an organism's survival is the ability to mount a robust transcriptional response to the imposed stress. An emerging mechanism of transcriptional control involves dynamic changes in chromatin structure. Alterations in chromatin structure are brought about by a number of different mechanisms, including chromatin modifications, which covalently modify histone proteins; incorporation of histone variants; and chromatin remodeling, which utilizes ATP hydrolysis to alter histone-DNA contacts. While considerable insight into the mechanisms of chromatin remodeling has been gained, the biological role of chromatin remodeling complexes beyond their function as regulators of cellular differentiation and development has remained poorly understood. Here, we provide genetic, biochemical, and biological evidence for the critical role of chromatin remodeling in mediating plant defense against specific biotic stresses. We found that the *Arabidopsis* SWI/SNF class chromatin remodeling ATPase SPLAYED (SYD) is required for the expression of selected genes downstream of the jasmonate (JA) and ethylene (ET) signaling pathways. SYD is also directly recruited to the promoters of several of these genes. Furthermore, we show that SYD is required for resistance against the necrotrophic pathogen *Botrytis cinerea* but not the biotrophic pathogen *Pseudomonas syringae*. These findings demonstrate not only that chromatin remodeling is required for selective pathogen resistance, but also that chromatin remodelers such as SYD can regulate specific pathways within biotic stress signaling networks.

## Author Summary

In eukaryotes, genomic DNA is organized into a complex DNA-protein structure termed chromatin. The organization of chromatin serves to compact DNA within the nucleus and plays a central role in regulating transcription by controlling the access of DNA to transcriptional machinery. Chromatin structure can be altered through several mechanisms, one of which is chromatin remodeling, a process that disrupts DNA–protein interactions resulting in altered accessibility of specific DNA regions to regulatory proteins in the transcriptional machinery. In this study, we investigated the biological role of chromatin remodeling in defense responses to biotic stresses using the model plant *Arabidopsis*. We found that a chromatin remodeling protein, SPLAYED, is required for gene expression within specific biotic stress signaling networks. Consistent with this observation, loss of SPLAYED chromatin-remodeling activity resulted in increased susceptibility to a fungal pathogen, *Botrytis cinerea*, but not to a bacterial pathogen, *Pseudomonas syringae*. These results demonstrate that reduced stress tolerance in a chromatin-remodeling mutant plant can be stress specific, and is not simply due to a decrease in overall fitness as a result of non-specific global mis-regulation of gene expression.

## Introduction

In eukaryotic organisms genomic DNA is packaged into chromatin, which can repress transcription by blocking the access of regulatory proteins to DNA. Dynamic changes in chromatin structure are now recognized as a robust mechanism of transcriptional control [1]–[3]. Changes in chromatin structure are brought about by a number of different mechanisms including: chromatin modifications, which covalently modify histone proteins; incorporation of histone variants; and chromatin remodeling, which utilizes ATP hydrolysis to alter histone-DNA contacts [1], [3]–[5]. ATP-dependent chromatin remodeling complexes are present in all eukaryotic organisms and can be grouped into three main classes: the SWI/SNF ATPases, the imitation switch (ISWI) ATPases, and the chromodomain and helicase-like domain (CHD) ATPases [2],[3].

Significant advances have been made in understanding the mechanism of ATP-dependent chromatin remodeling complex action [1],[4]. However, the biological role of chromatin remodeling complexes remains poorly understood, particularly in multicellular organisms where null mutations tend to be lethal [3],[6]. Studies that have investigated the biological role of chromatin remodeling complexes in multicellular organisms have largely focused on their role as regulators of cellular differentiation and development [2],[3]. In particular, *Arabidopsis* has served as a valuable model due to the fact that mutants in genes encoding a number of chromatin remodeling complex proteins are viable. One of the most well characterized chromatin remodeling complex proteins in *Arabidopsis* is the SWI/SNF class chromatin remodeling ATPase SPLAYED (SYD). Loss of SYD activity causes defects in many different developmental pathways including stem cell maintenance, patterning, developmental transitions and growth [3], [7]–[9].

The biological role of altering chromatin structure in response to stress via chromatin modifications and incorporation of histone variants has been documented [10]–[14]. However, the biological role of chromatin remodeling complexes or their specificity remains poorly understood. The role of chromatin remodeling in response to stress has been best studied in yeast where it has been shown that chromatin remodeling complexes are required for stress tolerance and are recruited to specific promoters upon stress [15]–[19]. However, few studies performed in multicellular organisms have investigated the role of chromatin remodeling in mediating stress responses. One study conducted in the human cell culture line SW480 demonstrated that chromatin remodeling complexes are recruited to specific promoters upon oxidative stress, which suggests that chromatin remodeling plays a role in the stress tolerance of multicellular organisms [20]. Additionally, it is unknown in any eukaryotic organism whether reduced stress tolerance in chromatin remodeling mutants is stress specific or indicative of decreased overall fitness due to non-specific global mis-regulation of gene expression.

In this study we examine the role chromatin remodeling plays in biotic stress responses. We found that SYD is required for expression of specific genes within biotic stress signaling networks. This requirement is likely both direct and indirect as SYD is recruited to the promoter of some, but not all, of the genes for which it is required for expression. We show that SYD is required for resistance against the necrotrophic pathogen *B. cinerea* but not the biotrophic pathogen *P. syringae*. These findings demonstrate not only that chromatin remodeling is required for selective pathogen resistance, but also that chromatin remodelers, such as SYD can regulate specific pathways within biotic stress signaling networks.

## Results/Discussion

**Figure 1:**
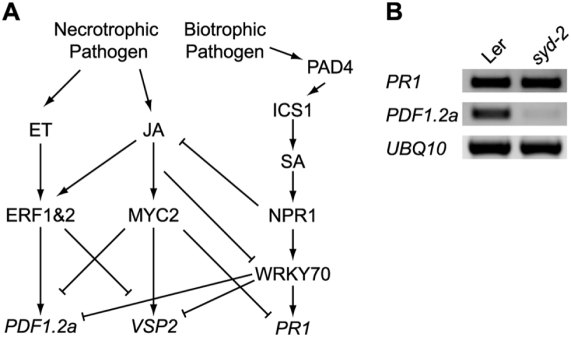
SYD is required for ET and JA marker gene expression. (A) Simplified model of the stress responsive network involving ET, JA, and SA signaling pathways [22],[23]. (B) RT-PCR analysis of basal levels of SA responsive *PR1* and ET and JA responsive *PDF1.2a* expression in WT (Ler) and *syd-2* rosette leaves.

We investigated the role of chromatin remodeling in stress signaling using *Arabidopsis*, a multicellular organism where viable chromatin remodeling null mutants exist [9]. In a previous study we showed that the SWI/SNF class chromatin remodeling ATPase *SYD* transcript is upregulated rapidly following mechanical wounding [21]. We also demonstrated that mechanical wounding is a response common to numerous biotic stresses that a plant may encounter [21]. The upregulation of *SYD* in response to wounding suggests that SYD may be recruited to remodel promoters within stress signaling networks. To begin delineating the placement of SYD in stress signaling we first examined whether SYD is required for expression of other transcripts upregulated rapidly in response to wounding. This demonstrated that SYD is not required for the expression of the rapid wound response genes *ETHYLENE RESPONSE FACTOR #18* (*ERF#18*) or *CCR4 ASSOCIATED FACTOR 1-like* (*CAF1-like*) (Figure S1A). We next investigated the role of SYD in the ethylene (ET), jasmonate (JA), and salicylic acid (SA) stress signaling pathways, which respond to abiotic and biotic stresses such as wounding and pathogen infection (Figure 1A) [22],[23]. As shown in Figure 1B, basal expression of the plant defensin *PDF1.2a*, a marker for intact ET and JA signaling, is lost in *syd-2* null mutants [9]. As basal levels of *PDF1.2a* are generally low, but detectable, we increased cycle number to improve our ability to detect basal differences between *syd-2* and wild-type (WT) [24],[25]. In contrast, basal expression of *PATHOGENESIS-RELATED1* (*PR1*), a marker for intact SA signaling, is maintained in *syd-2* plants (Figure 1B). These data suggest that SYD is required for ET and JA signaling but not SA signaling.

**Figure 2:**
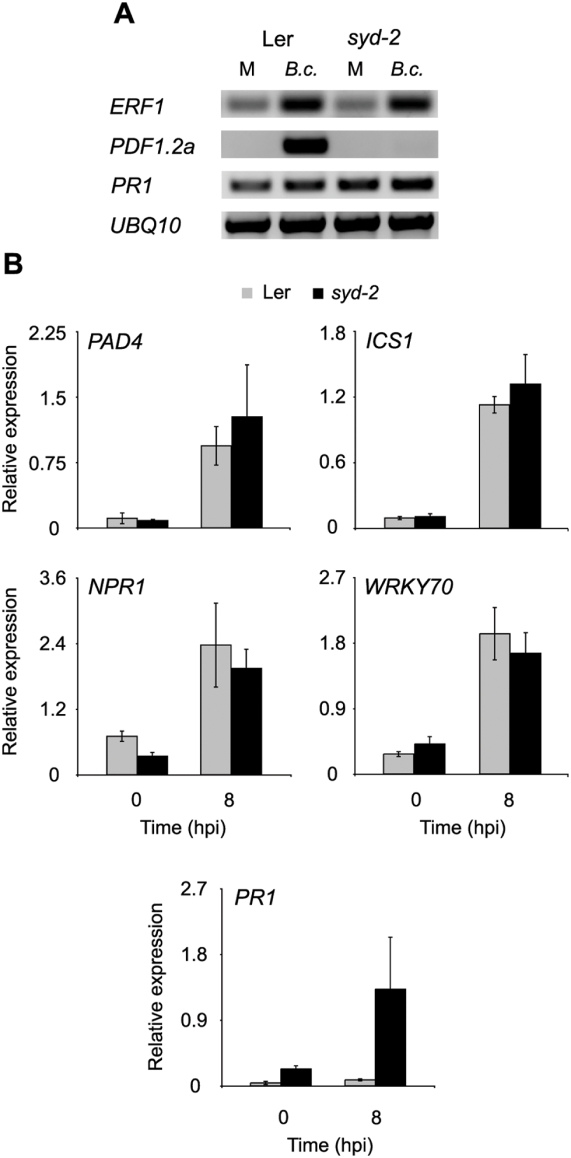
SYD is required within specific stress signaling pathways in response to biotic stress. (A) RT-PCR analysis of select ET/JA responsive genes in Ler and *syd-2* detached rosette leaves either mock (M) or *B. cinerea* (*B.c.*) treated for 48 h. (B) RT-qPCR analysis of SA biosynthesis and signaling genes 0 and 8 hours post inoculation (hpi) with 2×10^8^ CFU/ml virulent *P. syringae* pv. tomato (Pst) DC3000. Transcript levels were normalized to internal control genes measured in the same samples. Data are means of 3 independent biological replicates ±SEM.

The loss of basal *PDF1.2a* but not *PR1* expression in non-stressed *syd-2* plants suggests that SYD impacts specific stress signaling pathways. To explore the role of SYD under inductive stress treatments we inoculated plants with the necrotrophic pathogen *Botrytis cinerea* and the virulent biotrophic pathogen *Pseudomonas syringae*. As resistance to *B. cinerea* requires ET and JA signaling, whereas resistance to *P. syringae* is predominantly mediated by SA signaling [22],[26], use of these two pathogens allows further experimental evaluation of requirements for SYD function in defense signaling. We first monitored expression of key genes in the ET/JA pathway in response to *B. cinerea* treatment. The expression of the transcription factor *ETHYLENE RESPONSE FACTOR1* (*ERF1*), which requires both ET and JA for induction [22], [27]–[29], is similar in WT and *syd-2* plants (Figure 2A). In contrast to *ERF1*, the expression of *PDF1.2a* requires SYD in response to *B. cinerea* (Figure 2A). In addition, we examined the expression of *PR1* in plants treated with *B. cinerea* and determined that this gene is expressed at similar levels in WT and *syd-2* plants (Figure 2A). We next assayed the expression of a suite of genes involved in SA biosynthesis and signaling in response to *P. syringae* and found that SYD is not required for their expression (Figure 2B and Figure S2). Additionally, expression of *PR1*, but not upstream genes (*PAD4*, *ICS1*, *NPR1*, and *WRKY70*), is enhanced in *syd-2* plants. The apparent lack of detectable enhancement in *PR1* expression levels by RT-PCR (Figure 1B) is likely due to signal saturation inherent to ETBr staining. However, this pattern of transcriptional alteration is similar to what is observed in *myc2/jin1* mutants, suggesting that *MYC2* expression may be reduced in *syd* (Figure 1A) [22],[30]. Furthermore, as SWI/SNF class chromatin remodeling ATPase's are primarily considered activators of transcription it is highly unlikely that SYD is acting directly to repress *PR1* expression in WT plants [4]. Taken together these data demonstrate that SYD is required within specific stress signaling pathways in response to pathogen infection.

**Figure 3:**
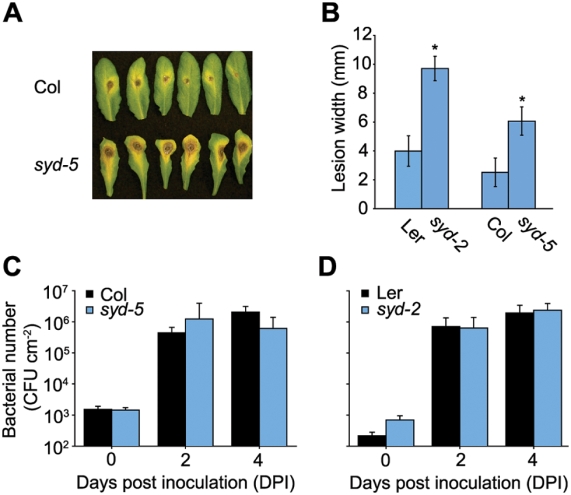
The chromatin remodeling ATPase SYD is required for resistance to *B. cinerea*. (A) Visual symptoms 4 d following spot inoculation with *B. cinerea* spores. (B) Lesion size 4 d after spot inoculation with *B. cinerea* spores. Data are means of 16 independent biological replicates ±SEM. Asterisks denote a significant difference from WT (*P*<0.05) as determined by *t*-tests. *B. cinerea* susceptibility assays were performed 3 times with similar results. Statistically different lesion size was also observed 3 d after inoculation. (C,D) Bacterial growth in WT and *syd* inoculated with 2×10^4^ CFU/ml virulent *P. syringae* pv. tomato (Pst) DC3000. Data are means of 8 independent biological replicates ±SD. No significant differences were detected by *t*-tests. Pathogen assays comparing Ler versus *syd-2* were repeated 3 times with similar results.

The finding that SYD is required for gene expression within specific stress signaling pathways suggests that loss of SYD function may reduce tolerance to specific biotic stresses. To test this hypothesis we first examined the resistance of *syd* mutants to *B. cinerea*. For this experiment we tested two independent *syd* null alleles and compared them to their respective WT background. As shown in Figure 3A and 3B, *syd* mutant plants are more susceptible to *B. cinerea* infection. The increased susceptibility of the *syd* mutants to *B. cinerea* is likely due to altered ET and/or JA signaling impacting defence mechanisms. It should also be noted that the phytoalexin camalexin plays a role in *B. cinerea* resistance [26]. However no significant difference in camalexin levels were detected between *syd* mutant and WT plants after elicitor treatment (data not shown). This suggests that SYD affects *B. cinerea* resistance through ET/JA signaling independent of camalexin production. To determine if reduced resistance was specific to *B. cinerea* we inoculated *syd* mutants with virulent *P. syringae*, for which resistance is predominantly mediated via SA signaling [26]. In contrast to *B. cinerea*, *syd* mutants and WT plants show similar resistance to *P. syringae* (Figure 3C and 3D). These results demonstrate that chromatin remodeling via SYD is required for stress specific disease resistance.

**Figure 4:**
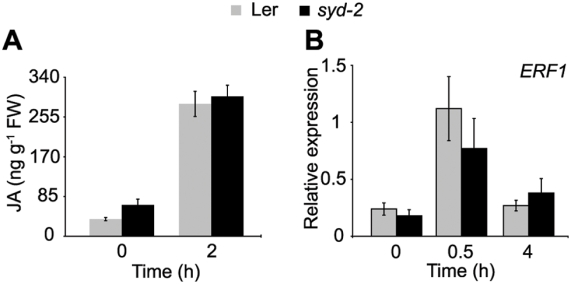
SYD activity is required downstream of ET and JA biosynthesis. (A) Measurement of JA metabolite levels in non-wounded and wounded Ler and *syd-2* plants. Data are means of 3 independent biological replicates ±SD. (B) Total RNA was extracted from non-wounded and mechanically wounded rosette leaves and subjected to RT-qPCR analysis. *ERF1* transcript levels were normalized to *At4g26410* measured in the same samples. Data are means of 3 independent biological replicates ±SEM.

SYD was originally implicated in stress responses by the observation that *SYD* transcripts accumulate upon wounding. [21]. We therefore examined which aspects of the ET and JA signaling pathways are impacted in *syd* mutants following wounding. Furthermore, we wished to directly compare gene expression levels with SYD recruitment to specific promoters via chromatin immunoprecipitation (ChIP) assays. Wounding is therefore advantageous as it enables better synchronization of the stress stimulus and is a feasible treatment for the large amount of tissue required for ChIP. We first monitored the expression of *ALLENE OXIDE SYNTHASE*, which is involved in JA biosynthesis, and found its transcription to be similar in WT and *syd-2* plants before and after wounding (Figure S1B). Measurement of JA levels reveals that basal and wound-induced JA biosynthesis is intact in *syd-2* plants (Figure 4A). In agreement with *B. cinerea* treatment (Figure 2A), expression of *ERF1* is similar in WT and *syd-2* in response to wounding (Figure 4B). Furthermore, an additional ethylene response factor (*ERF2*), which when overexpressed results in enhanced *PDF1.2a* levels [31], was similar in WT and *syd-2* (Figure S1B). These data collectively indicate that SYD activity is required downstream of ET and JA biosynthesis and *ERF1&2* expression.

**Figure 5:**
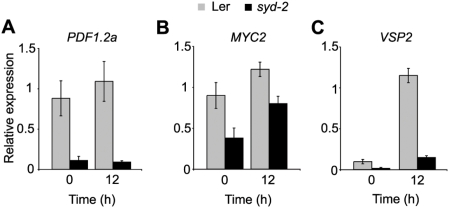
SYD regulates expression of ET and JA responsive defense genes. (A–C) Total RNA extracted from non-wounded and wounded Ler and *syd-2* rosette leaves was subjected to RT-qPCR analysis. Transcript levels were normalized to both *At4g34270* and *At4g26410* measured in the same samples. Data are means of 3 independent biological replicates ±SEM.

Downstream of *ERF1* the expression of *PDF1.2a* is severely reduced, to similar levels, before and after wounding in *syd-2* mutants (Figure 5A). SYD is also required downstream of JA biosynthesis for the expression of the bHLH Leu zipper transcription factor *MYC2* (Figure 5B). The reduced expression of *MYC2* suggests that the increased level of *PR1* may indeed be due to decreased *MYC2* levels in *syd* plants. Consistent with the reduced level of *MYC2* transcripts, the expression of *VEGETATIVE STORAGE PROTEIN* 2 (*VSP2*), a gene in the JA signaling pathway which requires *MYC2* for expression [22], [32]–[35], is severely reduced in *syd-2* mutants (Figure 5C). Taken together these data show that while chromatin remodeling via SYD is not required for expression of ET and JA biosynthesis genes, SYD activity is required for expression of *PDF1.2a*, *MYC2* and *VSP2*.

**Figure 6:**
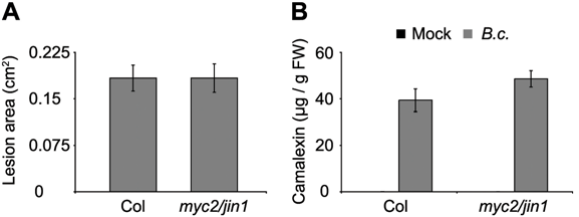
Role of MYC2 in response to *B. cinerea*. (A) Lesion size 3 d after spot inoculation with *B. cinerea* spores. (B) Camalexin levels of detached rosette leaves either mock (M; black bar) or *B. cinerea* (*B.c.*; grey bar) treated for 3 d. Results for *B. cinerea* isolate Grape are shown. Two alleles were tested, *jin1-8* and *jin1-9* [33],[34], and no significant differences between alleles were detected. We therefore pooled the two alleles to increase statistical power and refer to them as *myc2/jin1*. Data are means of 8 (Col) or 16 (*myc2/jin1*) independent biological replicates ±SEM. Experiments were independently repeated with similar results.

The finding that *MYC2* transcript levels are reduced in *syd-2* plants (Figure 5B), even though *syd-2* is more susceptible to *B. cinerea* (Figure 3A and 3B), appears to conflict with published models of defense signaling where MYC2 acts as a negative regulator of *PDF1.2a* expression and resistance to necrotrophic pathogens (Figure 1A) [22],[34]. However, the apparent discrepancy can be reconciled. The slight increase in resistance against *B. cinerea* of *myc2/jin1* mutants is thought to be due to derepression of pathogen defense genes such as *PDF1.2a* [34]. In *syd-2* mutants derepression of *PDF1.2a* does not occur even though the level of *MYC2* is reduced, suggesting that the requirement of SYD for *PDF1.2a* expression precedes repression of *PDF1.2a* by *MYC2*. It is also possible that the level of *MYC2* transcript reduction in *syd-2* is not great enough to have a measurable biological impact. Additionally, increases in resistance to *B. cinerea* exhibited by the *myc2/jin1* mutants, assayed by qualitative disease symptom rating, appeared to be subtle [34], suggesting that the *myc2/jin1* effect could be masked in *syd* mutants. To better quantify the impact of MYC2 on resistance to *B. cinerea*, we measured lesion development on leaves of *myc2/jin1* mutant plants following infection with multiple *B. cinerea* isolates. We found no significant quantitative difference in lesion formation between WT and *myc2/jin1* mutants (Figure 6A and Figure S3A and S3C). We also measured defense-associated secondary metabolites, including camalexin and glucosinolates, in mock- and *B. cinerea-* treated WT and *myc2/jin1* plants. Glucosinolates are associated with *Arabidopsis* defense against insect herbivores and pathogens and some are regulated by JA signaling [36]. Of the five measured metabolites known to be regulated by JA, camalexin and indole-3-yl-methyl were unaffected by the *myc2/jin1* mutation in comparison to WT, 3-methylsulfinyl and 4-methylsulfinyl decreased only in mock treated *myc2/jin1* while 4-methoxy-indole-3-yl-methyl was present at higher concentrations in only *B.cinerea* treated *myc2/jin1* (Figure 6B and Figure S3B, S3D and S3E and Table S1). Together these data suggest that MYC2 has neither directionally consistent nor major impacts on all molecular JA responses. It is therefore not surprising that *syd* mutants are more susceptible to *B. cinerea* even though *MYC2* levels are reduced in *syd-2*.

**Figure 7:**
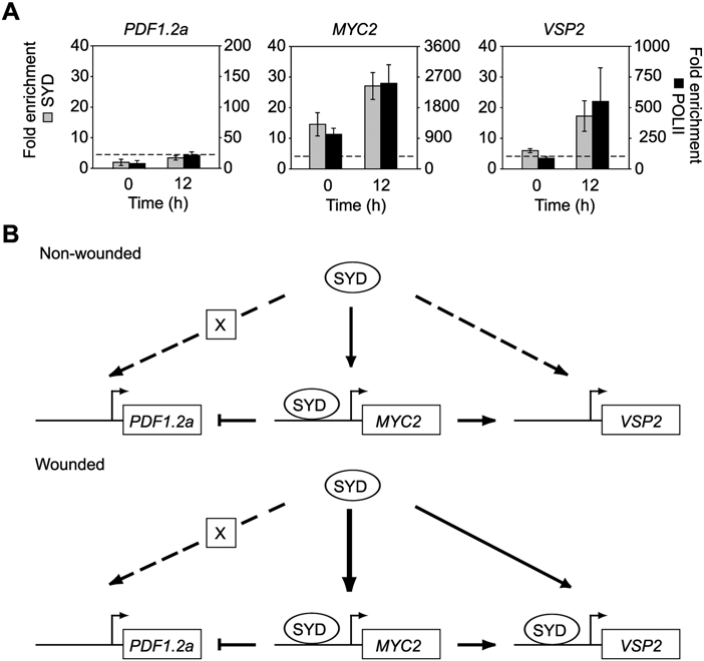
SYD is recruited to the promoters of ET and JA responsive defense genes. (A) ChIP-qPCR analysis of SYD (grey bars; left y-axis) and POLII (black bars; right axis) recruitment to the promoters of *PDF1.2a* (−323 to −151), *MYC2* (−320 to −222), and *VSP2* (−226 to −147). Data presented are normalized to input DNA and expressed as fold enrichment of SYD or POLII relative to IgG. ChIP-qPCR was performed on non-wounded and wounded Ler plants. Data are means of 3 or 4 independent biological replicates ±SEM. The dashed line represents the mean SYD background fold enrichment at non-specific genomic loci assayed at the seed specific promoters of *OLEOSIN1* and *AT2S3*. (B) Model summarizing the roles of SYD in response to wounding.

The requirement of SYD for the expression of select ET and JA responsive genes suggests that SYD may be directly recruited to remodel their promoter regions, thereby enabling transcriptional induction. To test this hypothesis *in vivo* we performed ChIP followed by quantitative polymerase chain reaction (ChIP-qPCR) assays using the SYD specific antibody, which was previously used in ChIP experiments to show that SYD binds the *WUSCHEL* promoter to regulate stem cell fate [8]. Additionally, ChIP-qPCR was performed using IgG (negative control) and RNA polymerase II (POLII) (positive control for actively transcribed regions) antibodies. The background level of SYD binding to non-specific genomic loci (dashed line in Figure 7A and Figure S4) was determined by ChIP-qPCR performed on the promoters of two seed specific genes, *OLEOSIN1* and *AT2S3*, which are subject to repressive histone H3 lysine 27 trimethylation and are not expressed in *Arabidopsis* leaf tissue [37]. Additionally, ChIP-qPCR was performed on *syd-2* tissue wounded for 12 h to further ensure that the results are SYD specific (Figure S5). Under the experimental conditions tested, SYD does not bind the promoter of either *PDF1.2a* or *ERF1* above the background level of detection (Figure 7A and Figure S4). To further verify the lack of SYD binding to *PDF1.2a* a second region of the *PDF1.a* promoter was assayed and SYD binding was not detected (Figure S4). As shown in Figure 7A, SYD binds the promoter of *MYC2* before and after wounding. Finally, SYD is recruited to the promoter of *VSP2* following wounding (Figure 7A).

Based on our findings we propose a model (Figure 7B) that summarizes the roles of SYD in response to wounding. Although SYD is required for the expression of *PDF1.2a* we were unable to detect SYD enrichment at the promoter of *PDF1.2a*, suggesting that SYD may act indirectly through an unknown factor(s) to enable transcription of *PDF1.2a*. Additionally, SYD is bound to the *MYC2* promoter, which is consistent with the reduced expression of *MYC2* in *syd-2*. The direct recruitment of SYD to the *MYC2* promoter may also help explain the reduced transcript levels of *VSP2*, in non-wounded *syd-2*, even though SYD binding to the *VSP2* promoter region was only detected following wounding. Altogether these data suggest that the altered expression of ET and JA responsive genes in *syd-2* is likely a result of the loss of SYD acting both directly and/or indirectly on their promoters to regulate transcription.

### Conclusions

Our results show that ATP-dependent chromatin remodeling is required for expression of specific genes within stress signaling networks. Additionally, this requirement is likely both direct and indirect as the chromatin remodeling ATPase SYD binds several, but not all, of the stress responsive promoters examined *in vivo*. Loss of chromatin remodeling activity also results in increased susceptibility to *B. cinerea* but not *P. syringae*. These results provide biological evidence that chromatin remodeling complexes, which are evolutionarily conserved within eukaryotes, are required for stress tolerance not only in yeast but also multicellular organisms. Furthermore, the requirement of ATP-dependent chromatin remodeling complexes is pathogen-specific and not a result of a general reduction in fitness.

## Materials and Methods

### Plant growth conditions and treatment

*Arabidopsis thaliana* plants were grown in a 16 h light/8 h dark photoperiod at 22°C; except plants for pathogen treatments, which were grown in a 12 h light/12 h dark photoperiod. Wounding was performed as previously described [21]. All experiments were performed on 4 to 5-wk-old plants, which exhibited no disease symptoms or insect herbivory prior to treatment. Detached leaf assays were performed using the *B. cinerea* isolates DN, Grape, B05.10 and 83-2 [38]. *Arabidopsis* leaves were inoculated with 5 µl of spores at a concentration of 50,000 spores/ml [38],[39]. For *P. syringae* bacterial growth assays *Arabidopsis* leaves were inoculated with 2×10^4^ CFU/ml *P. syringae* pv. tomato (Pst) DC3000 by hand injection.

### Expression analyses

Total RNA from rosette leaves was isolated by TRIzol extraction (Life Technologies, Grand Island, NY) and treated with DNAaseI to control for DNA contamination. RNA was reverse transcribed using Superscript III (Invitrogen, Carlsbad, California). PCR for RT-PCR were conducted in 25 µl reactions containing 20 ng cDNA, 1.5 mM MgCl_2_, 0.2 mM each dNTP, 0.05 µM each primer, and 1 U Choice-Taq Blue (Denville Scientific, Metuchen, NJ) and amplified for 29 cycles except for *PDF1.2a* in Figure 1B and *ERF1* in Figure 2A, which were amplified for 34 cycles. Quantitative RT-PCR was conducted in 50 µl reactions containing 10 ng cDNA, 1× iQ SYBR Green supermix (Bio-Rad Laboratories, Hercules, CA), and 200 or 250 nM each primer. Amplification and data analysis were carried out as previously described [21]. The internal controls *At4g34270* and *At4g26410* previously described were used for transcript normalization [40]. Primers are listed in Table S2.

### JA measurement

Extraction of JAs (MeJA and JA) were carried out as previously described [41] and further analyzed by GC-MS using a Hewlett and Packard 6890 series gas chromatograph coupled to an Agilent Technologies 5973 network mass selective detector operated in electronic ionization (EI) mode.

### Camalexin and glucosinolate measurement

Camalexin and glucosinolates were measured 72 h after mock or *B. cinerea* inoculation as previously described [42]. Briefly, individual leaves were collected into deep 96-well plates containing 0.5 ml 90% methanol in each well. Following tissue disruption and centrifugation, 150 µl of leaf extract was removed for camalexin measurement. De-sulfo glucosinolates were extracted from an additional 150 µl of the same sample by passing the methanolic extract over a column of DEAE Sephadex A-25 (Sigma-Aldrich) and, after methanol and water washes, incubating the samples overnight with an excess of sulfatase before eluting with 150 µl H_2_0. Extractions were performed largely as previously described, but using centrifugation rather than vacuum to remove liquid from the Sephadex columns [43]. Separation of 50 µl of aqueous extracts was performed on a 5-µm column (Lichrocart 250-4 RP18e, Hewlett-Packard, Waldbronn, Germany) attached to a Hewlett-Packard 1100 series HPLC, using the following series of solvent gradients: 6-min 1.5% to 5.0% (v/v) acetonitrile, 2-min 5% to 7% (v/v) acetonitrile, 7-min 7% to 25% (v/v) acetonitrile, 2-min gradient from 25% to 92% (v/v) acetonitrile, 6 min at 92% (v/v) acetonitrile, 1-min 92% to 1.5% (v/v) acetonitrile, and a final 5 min at 1.5% (v/v) acetonitrile. Compounds were detected at 229 nm using a diode array detector, identified by comparison with retention time and absorption spectra of purified references, and quantified using response factors as previously published (Table S1) [44],[45].

### ChIP-qPCR

ChIP-qPCR assays were performed as previously described [8] with the following modifications. Each ChIP was conducted using 500 mg of Ler rosette leaf tissue. DNA was sonicated to a size range of 0.3–1.5 kb. For the IgG control ChIP 2 µg of IgG from rabbit serum (Sigma, St. Louis, MO) was used. Following reverse cross-linking of the immunoprecipitation reactions the samples were treated with RNase A solution (CalBiochem, La Jolla, CA) and Proteinase K (Sigma, St. Louis, MO). qPCR of the ChIP eluates was performed with iQ SYBR Green supermix according to manufacturer. ChIP-qPCR results were calculated based on the ΔΔC_t_ method using the SuperArray ChIP-qPCR Data Analysis Template (Frederick, MD) according to the SuperArray manual, as described [46]. Briefly, ChIP DNA fractions were first normalized to input DNA (ΔC_t_) to account for chromatin sample preparation differences. Input normalized SYD and POLII ChIP fractions were then adjusted for the normalized non-specific background (IgG) giving the ΔΔC_t_ value. Fold differences relative to the IgG reference were then calculated by raising 2 to the ΔΔC_t_ power. The primers used in this study are listed in Table S2.

### Statistical analysis

To determine statistical significance of treatment effects comparing WT versus *syd t-*tests were performed using Sigma Stat v3.5 (San Jose, CA). For comparison of WT versus *myc2/jin1* factorial ANOVA performed within SAS (Cary, NC) was used to analyze the effects of genotype and treatment on measured phenotypes, with significance of differences determined via t-tests of pre-selected comparisons.

### Accession numbers

*PR1*: At2g14610, *PDF1.2a*: At5g44420, *UBQ10*: At4g05320, *ERF1*: At3g23240, *PAD4*: At3g52430, *ICS1*: At1g74710, *NPR1*: At1g64280, *WRKY70*: At3g56400, *ERF#18*: At1g74930, *CAF1-like*: At3g44260, *AOS*: At5g42650, *ERF2*: At5g47220, *MYC2*: At1g32640, *VSP2*: At5t24770, *Oleo1*: At4g25140, *AT2S3*: At4g27160

## Supporting Information

Figure S1 Expression of wound responsive transcripts in *syd-2*. (A) RT-PCR analysis of select rapid wound response genes in Ler and *syd-2* plants in response to wounding (B) RT-PCR analysis of the JA biosynthesis gene *AOS* and the ET/JA responsive *ERF2* in response to wounding.
(0.65 MB EPS)

Figure S2 RT-PCR expression analysis of genes involved in SA biosynthesis and signaling in response to *P. syringae*.
(0.71 MB EPS)

Figure S3 Role of MYC2 in response to *B. cinerea*. (A) Lesion size 3 d after spot inoculation with *B. cinerea* isolate 83-2. (B) Camalexin levels of detached rosette leaves either mock (M; black bar) or *B. cinerea* isolate 83-2 (*B.c.*; grey bar) treated for 3 d. (A and B) Two alleles were tested, *jin1-8* and *jin1-9*. No significant differences between alleles were detected, therefore the two alleles were pooled (*myc2/jin1*). Data are means of 8 (Col) or 16 (*myc2/jin1*) independent biological replicates ±SEM. Experiments were repeated with similar results. (C) Lesion size 3 d after spot inoculation of Col and *jin1-9* plants with *B. cinerea* isolate B05.10. Data are means of 8 independent biological replicates ±SEM. (D) Camalexin levels of detached rosette leaves treated with *B. cinerea* isolate B05.10 for 3 d. Data are means of 8 independent biological replicates ±SEM. (E) Camalexin levels of detached rosette leaves treated with 5 mM AgNO_3_ for 3 d. Data are means of 8 independent biological replicates ±SEM.
(0.62 MB EPS)

Figure S4 SYD does not bind the promoter of *PDF1.2a* or *ERF1*. ChIP-qPCR analysis of SYD (grey bars; left y-axis) and POLII (black bars; right axis) recruitment to the promoter of (A) *PDF1.2a* (−836 to −604) and (B) *ERF1* (−1035 to −942). Data presented are normalized to input DNA and expressed as fold enrichment of SYD or POLII relative to IgG. ChIP-qPCR was performed on non-wounded and wounded Ler plants. Data are means of 3 or 4 independent biological replicates ±SEM. The dashed line represents the mean SYD background fold enrichment assayed at the promoters of *OLEOSIN1* and *AT2S3*.
(0.57 MB EPS)

Figure S5 Background levels of SYD ChIP-qPCR signal in *syd-2* plants. ChIP-qPCR analysis of SYD recruitment to the promoters of *PDF1.2a* (−323 to −151), *ERF1* (−1035 to −942), *MYC2* (−320 to −222), and *VSP2* (−226 to −147). Data are expressed as fold enrichment of SYD relative to IgG. ChIP-qPCR was performed on 12 h wounded Ler and *syd-2* plants. Data are means of 3 or 4 independent biological replicates ±SEM.
(0.47 MB EPS)

Table S1 Glucosinolate levels in *myc2/jin1* plants.
(0.03 MB XLS)

Table S2 Primers used for expression and ChIP-qPCR analysis.
(0.02 MB XLS)
